# Activities-specific performance frequency can accurately detect fallers in elderly populations: an alternative method for quantifying activity restrictions

**DOI:** 10.1186/s12877-022-02912-z

**Published:** 2022-03-14

**Authors:** Lin Y. Chen, Jing X. Wang, Ying Y. Chen, Ya J. Yang, Jia J. Yao, Xia Shen

**Affiliations:** 1 Shanghai YangZhi Rehabilitation Hospital (Shanghai Sunshine Rehabilitation Center), Tongji University School of Medicine, Shanghai, 201619 China; 2 Department of Rehabilitation Sciences, Tongji University School of Medicine, Shanghai, 200092 China

**Keywords:** falling, elderly, activity restriction, balance, mobility

## Abstract

**Background:**

The high prevalence of falling among older adults constitutes a major public and clinical health concern. Many elderly persons may develop activities-specific restriction due to the risk of falling. This highlights the need for relevant evaluative tools.

**Methods:**

This cross-sectional study used activities-specific performance frequency indicators to quantify activity restrictions in elderly participants, with all measures based on items from the Activities-Specific Balance Confidence (ABC) scale. Specifically, we tested for correlations between activities-specific performance frequency and balance confidence, functional balance/mobility, and fall history. There were 88 elderly participants, including 28 with stroke, 30 with Parkinson’s disease, and 30 with no neurological diseases. In addition to their activities-specific performance frequency measures, we collected a series of demographic and health-related characteristics from each participant. We analyzed between-group differences in activities-specific performance frequency and other demographic and health-related characteristics via the one-way analysis of variance and Kruskal-Wallis test. Next, we used the Spearman’s rank correlation test and binary logistic regression to investigate the correlations between activities-specific performance frequency and demographic/other health-related characteristics.

**Results:**

There were significant group differences in performance frequency for all ABC activity items except for walking around the house, average ABC scores, and functional balance/mobility among normal older adults, participants with strokes and those with Parkinson's disease. Activities-specific performance frequency showed stronger correlations with activities-relevant functional mobility (*r*=0.250-0.713 for 15 items with significant correlations, 13 activity items with *r*≧0.4) than with balance confidence (*r*=0.279-0.668 for 13 items with significant correlations, 10 activity items with *r*≧0.4). The performance frequency of walking in crowds/bumped was the most sensitive measure for predicting fallers (odd ratio=3.310, *p*<0.05).

**Conclusions:**

This study proposed and validated the usage of activities-specific performance frequency as an alternative method for quantifying activity restrictions among older adults.

**Supplementary Information:**

The online version contains supplementary material available at 10.1186/s12877-022-02912-z.

## Background

For older adults, the high prevalence of falling has become a major concern in public and clinical health [1, 2], especially due to the significant impacts on health outcomes and quality of life, the associated treatment costs, and increased utilization of health care services [3, 4]. Research has shown that both physical inactivity and activity restriction are two main physical behavior related risk factors for falling among older adults [5]. More specifically, physical inactivity refers to a lifestyle in which an individual fails to engage in weekly minimums of either 150 minutes of moderate-intensity aerobic physical activity, 75 minutes of vigorous-intensity aerobic physical activity, or an equivalent combination of moderate and vigorous-intensity activities [6]. Meanwhile, activity restriction is typically used as a general term to describe reductions in normal daily activities [5]. Both conditions are thought to reduce mobility, more reduction indicating higher risk of falling [5, 7].

Unlike physical inactivity, activity restriction plays a dual role in fall prevention; here, an individual may initially or purposefully avoid falling risks by decreasing their exposure, but this eventually leads to excessive activities-specific fears of falling and deteriorated functional mobility [5, 8]. Activity restriction first occurs in the context of specific activities, including those that present environmental hazards (e.g., walking on slippery surfaces) and/or require complex mobility or endurance (e.g., visiting crowded locations or walking several blocks outside) [5]. In the long term, such restrictions may encroach into daily activity performance, thus increasing the overall risk of falling.

To our knowledge, no previous studies have empirically investigated how activities-specific restrictions impact the risk of falling. In general, this may be due to the lack of available tools, as few are designed to measure activities-specific restrictions or activities-specific performance frequency/volume. While the Survey of Activities and Fear of Falling in the Elderly (SAFE) is commonly used to assess activities-specific restrictions [5, 9],the tool only tests for activity restrictions at the time of application based on comparisons with the preceding five-year period, which is insufficient for tracking long-term changes in frequency and volume. While the more recently developed composite activities-specific risk of falls scale (CARFS) quantifies activity restrictions based on performance frequency and estimates the risk of falling in conjunction with the fear of falling [8], its psychometric properties have not been sufficiently investigated.

To address this gap, this study aimed to test the validity of using activities-specific performance frequency to assess falling risks in older adults. In this context, we focused on the correlations between activities-specific performance frequency and balance confidence, functional balance/mobility, and fall history. To obtain data, we designed a survey question on activities-specific performance frequency based on the CARFS, but adopted the activity item structure used in the Activities-Specific Balance Confidence (ABC) scale, which covers a series of activities across a wide difficulty range and has previously been shown to have good psychometric properties for use among the ambulatory older adults [10].

## Methods

### Design

This cross-sectional study investigated older participants using a series of questionnaires and physical assessments during a one-time visit. Prior to this, we assessed the test-retest reliability of our ABC-structured query on activities-specific performance frequency through two surveys among 10 elderly participants. The study was performed in accordance with the Declaration of Helsinki and was approved by the ethics committee of Shanghai Yangzhi Rehabilitation Hospital (Shanghai Sunshine Rehabilitation Center) (YZ2019-005) and the ethics committee of Shanghai Tongji Hospital (2020-062).

### Participants

There were 88 elderly participants, including 28 with stroke, 30 with Parkinson’s disease (PD), and 30 with no neurological diseases. Those with neurological diseases were recruited from the outpatient department at Shanghai Yangzhi Rehabilitation Hospital (Shanghai Sunshine Rehabilitation Center) and at Shanghai Tongji Hospital and through online WeChat groups designed for patients. Meanwhile, those with no neurological diseases were recruited by inviting the companions of patient study participants and searching for potential participants in communities located around respective hospitals. The inclusion criteria were as follows: 1) aged 55 years or older, 2) without any diagnosed neurological diseases or only diagnosed with stroke/PD, and 3) willing to participate and signing the informed consent. Persons who had communication problems or a score for the Mini-Mental State Examination test of 23 or lower, were excluded [11]. The 10 community-dwelling elderly persons who engaged in the test-retest component prior to the cross-sectional survey met the same selection criteria.

### Measures

All data were collected by two researchers who had sufficient training and could conduct all assessments reliably. The collected data consisted of basic demographic information, health conditions, activities-specific performance frequency based on the ABC activity items (Appendix I), overall daily sitting time, fall history within the past six months, activities-specific balance confidence measured by the ABC scale, and functional balance/mobility assessed via the functional reach (FR) test, timed up and go (TUG) test, and Functional Assessment Measure (FAM).

Basic demographic information included age and sex, while health information included whether the respondent was healthy or with Parkinson’s disease/stroke, followed by the time since Parkinson’s disease/stroke diagnosis and the side of hemiparesis in cases of stroke. The remaining components are described in further detail below.

### Activities-specific performance frequency

The ABC scale contains 16 items covering different functional activities [10]. For each activity, we presented participants with the prompt, “how often did you perform the following activities during the previous one-month period;” here, a 5-point Likert scale was extracted from the CARFS to measure activities-specific performance frequency over the designated one-month period [8], in which 0 indicated no engagement, 1 indicated occasional engagement, 2 indicated sometimes (weekly), 3 indicated often (daily), and 4 indicated very often (daily, but with a higher frequency than normal). The test-retest component of this research indicated high reliability for performance frequency in each assessed activity (Pearson correlation= 0.749-1.000).

### Sitting time item on the Physical Activity Scale for the Elderly

Most elderly persons are physically inactive; in other words, they spend most of their time in sedentary behaviors [12]. Accordingly, to minimize the survey load, we assessed sedentary behaviors solely based on sitting, which is the most common of such behaviors. Specifically, we used the sitting time item from the Physical Activity Scale for the Elderly [13], as follows: “Over the past 7 days, how many hours per day on average did you engage in sitting activities such as watching TV/computer/cellphone, reading, or doing handcrafts, paperwork, playing cards, sewing, etc.” We recorded the sitting time using the scale score, which ranged from 1 to 4 (1=less than 1 hour; 2=1–2 hours; 3=2–4 hours; 4=over 4 hours).

### Fall history

For fall history, participants were asked the following question: “Have you fallen within the past six months, and if yes, how many times?” Here, a fall was defined as an unexpected event through which an individual came to rest on the ground or some lower level, but not as the result of a major intrinsic event such as a syncope, stroke, seizure, or overwhelming hazard. Participants were classified as non-fallers, single fallers, and recurrent fallers if they respectively answered no, yes (one fall), and yes (more than one fall) to the above question [14].

### Activities-specific balance confidence

The ABC was used to measure activities-specific balance confidence, as it has been shown to have good psychometric properties for use among patients with stroke/PD and older adults in general [10, 15, 16]. Participants were asked to rate their confidence in performing each activity without losing balance by selecting a respective value between 0% (no confidence) and 100% (completely confident).

### Functional balance and mobility

The FR test assesses functional balance using one simple task. It exhibits excellent reliability for use among patients with stroke/PD and older adults in general [17–19]. In this study, a measuring tape was mounted on the wall at a height of 150cm. Initially, each participant stood with their feet shoulder-width apart and one arm at a 90° angle in the forward direction. The first reading was taken at the tip of the middle finger. Following this, the participant was instructed to lean their body forward as far as possible without changing their base of support, then hold their furthest position for at least three seconds. They were not permitted to raise their heels during the test; otherwise, a retest was initiated. The second reading was taken using the same reference point at the furthest position. For each participant, we calculated FR distance based on the number achieved after subtracting the second reading from the first reading. We allowed all participants to perform the test using their dominant or non-paretic side.

The TUG test is a simple way to measure functional mobility. In this study, participants were required to complete the following sequence of motions after being seated in an armchair: rise from the armchair, walk three meters forward, turn, walk toward the original position, and sit down in the same armchair. We recorded the time each participant needed to complete this procedure. Considering the applicability of the TUG test for all participants (as some of them could not perform the test owing to mobility dependence), participants’ performance in the test was categorized into six levels: 0 indicates no ability to perform the test, while 1, 2, 3, 4, and 5 indicate that the TUG test was completed in >60 s, >30 s, >20 s, >10 s, and ≤10 s, respectively. Several previous studies have shown that the TUG test is reliable for use in this target population [20–22].

Walking independence levels were scaled with the FAM, as follows: no disability (complete independence in a timely, safe manner), slight disability (modified independence with extra time or assistive devices), moderate disability (dependence with supervision), and severe disability (dependence with assistance) [23].

### Analysis

We used IBM SPSS 21.0 for all statistical analyses. Specifically, we conducted a one-way analysis of variance (ANOVA) and Kruskal-Wallis test to respectively examine the differences in quantitative and qualitative measures between participant groups (i.e., Stroke, PD, and Normal). Next, we conducted an independent t-test and Mann-Whitney U test to investigate post-hoc between-group contrasts.

We used Spearman’s rank correlation to test for correlations between activities-specific performance frequency and demographic/other health-related characteristics. Five levels of correlation strength were ranked based on the correlation coefficient (r): very weak (*r*= 0.00-0.19), weak (*r*= 0.20-0.39), moderate (*r*= 0.40-0.59), strong (*r*= 0.60-0.79), and very strong (*r*= 0.80-1.00 ) [24].

We conducted a binary logistic regression to identify the discriminators of fall history based on all demographic and health-related characteristics. We then conducted univariate regression analysis to identify these potential predictors, with the significance level for inclusion in multivariate regression analysis determined at 0.1; predictors with this significance level entered into the multivariate regression model [25]. Here, we adopted a multivariate regression with backward stepwise selection to extract the final model through exclusion, with significance levels set to 0.05. Finally, we used the receiver operating characteristic (ROC) curve to further determine cutoff scores for the significant predictors of fall history. For each test, final significance levels were set to 0.05.

## Results

As mentioned, we investigated a total of 88 elderly participants, including 28 with stroke, 30 with PD, and 30 with no neurological diseases.

For those with stroke, there were eight females and 20 males, with an average time after stroke onset of 10.4 months; four walked independently, 10 walked using aids, eight walked with supervision, and six walked with assistance. Of these participants, six had fall histories within the previous six months, including three single fallers and three recurrent fallers.

For those with PD, there were 17 women and 13 men, with an average disease duration of 76.4 months. All walked independently, but 10 had fall histories within the previous six months, including seven single fallers and three recurrent fallers.

For those without neurological diseases, there were 20 women and 10 men. All walked independently, but seven had fall histories within the previous six months, including six single fallers and one recurrent faller.

Based on the above, there were significant differences in the age and sex ratios between participant groups (*p*<0.05). There were also significant intergroup differences in performance frequency for all ABC activity items except for walking around the house, as well as significant differences in total daily sitting time, average ABC scores, and functional balance/mobility as measured via FR, TUG, and FAM (*p*<0.05). Most intergroup differences for most measures were found between the stroke and normal groups rather than the PD and normal groups, except for total daily sitting time, which only showed a significant difference between the PD and normal groups (Table 1). There were no significant intergroup differences in the ratio of fallers (*p*>0.05).

**Table 1:** Demographic and health-related characteristics for each study group

	Elderly participants				Group difference		
	All (*n*=88)	Stroke (*n*=28)	PD (*n*=30)	Normal (*n*=30)	All	Stroke vs Normal	PD vs Normal
Age	67.8±6.4	64.5±6.6	67.7±5.5	70.8±5.6	*	*	*
Sex (Female: male)	45:43	8:20	17:13	20:10	*	*	NS
Disease duration (months)	-	10.4±13.6	76.4±52.3	0±0	*	*	*
Activities-specific frequency (0-4)^a^							
Walk around house	3(0-4)	3(1-4)	3(3-4)	3(3-4)	NS	NS	NS
Up and down stairs	2(0-4)	0.5(0-4)	3(1-4)	3(0-4)	*	*	NS
Pick up slipper	3(0-4)	1(0-4)	3(0-4)	3(2-3)	*	*	NS
Reach at eye level	3(0-4)	1(0-4)	3(2-4)	3(0-4)	*	*	NS
Reach on tiptoes	1.5(0-3)	0(0-2)	2(0-3)	2(0-3)	*	*	NS
Stand on chair to reach	1(0-3)	0(0-0)	1(0-3)	1(0-3)	*	*	NS
Sweep the floor	2(0-4)	0(0-3)	2(0-4)	3(0-4)	*	*	NS
Walk outside to nearby car	0.5(0-3)	0(0-1)	1(0-3)	1(0-3)	*	*	NS
Get in/out of car	1(0-4)	1(0-2)	2(0-4)	2(0-3)	*	*	NS
Walk across parking lot	1(0-4)	0(0-1)	1(0-4)	1(0-3)	*	*	NS
Up and down ramp	1(0-4)	0(0-4)	1(0-3)	2(0-3)	*	*	NS
Walk in crowded mall	0(0-3)	0(0-1)	1(0-3)	1(0-3)	*	*	NS
Walk in crowd/bumped	0(0-3)	0(0-0)	0(0-2)	0(0-3)	*	*	NS
Escalator holding rail	1(0-3)	0(0-1)	2(0-3)	1(0-3)	*	*	*
Escalator not holding rail	0(0-3)	0(0-0)	0(0-3)	1(0-3)	*	*	NS
Walk on icy sidewalks	1(0-4)	0(0-0)	1(0-4)	1(0-3)	*	*	NS
Sitting time (1-4)^a^	4(1-4)	4(2-4)	3(1-4)	4(3-4)	*	NS	*
Fall history					NS	NS	NS
Non-faller	65	22	20	23			
Single faller	16	3	7	6			
Recurrent faller	7	3	3	1			
ABC overall (0-100)	75.1±23.2	52.5±25.8	82.3±13.2	89.1±8.5	*	*	NS
FR (cm)	24.3±7.4	19.1±7.3	27.6±6.6	25.6±5.9	*	*	NS
TUG test (0-5) ^a^	4(0-5)	2(0-4)	4(3-5)	4(4-5)	*	*	NS
Walking ability					*	*	NS
Complete independence	64	4	30	30			
Modified independence	10	10	0	0			
With supervision	8	8	0	0			
With assistance	6	6	0	0			

*: *p*<0.05; NS: *P*>0.05

^a^: the data of activities-specific frequency is shown with median and the range in brackets.

The Spearman’s rank correlation analysis showed that demographic and health-related characteristics correlated with activities-specific performance frequency to various degrees. For example, age and sex were weakly correlated with the performance frequency of several activity items, including sweeping the floor, walking on icy sidewalks, with older and female participants showing higher performance frequency. Sitting time weakly and negatively correlated with performance frequency in three activity items, including reaching at eye level and on tiptoes as well as taking escalator while holding onto a railing (*r*= -0.261, -0.253, -0.233). Falling history within the previous six months weakly correlated only with performance frequency in walking in crowds/bumped (*r*= 0.252). FR distance weak-to-moderately correlated with performance frequency in 13 activity items (*r*= 0.311 to 0.517), but moderate correlations appeared for only three items. TUG test performance (*r*= 0.250 to 0.686) and FAM (*r*= 0.283 to 0.713) showed significant correlations with performance frequency in 15 activities (excluding walking around house); among these, 12 and 13 activities had moderate-to-strong correlations with TUG and FAM, respectively. Different from the results for activities-specific performance frequency, sitting time only weakly associated with fall history (*r*= 0.228) and overall activities-specific balance confidence (*r*= -0.220) (Table 2).

**Table 2:** Correlations between physical behaviors and demographic/other health-related characteristics

Correlations (Spearman’s rho)	Age (y)	Sex (M:1,F:2)	Sitting time (1-4)	Fall history (N:0, Y:1)	FR (cm)	TUG (0-5 levels)	Walking ability	Overall ABC (0-100)
Activities-specific frequency (0-4)								
1.Walk around house	.113	.014	-.022	-.055	-.106	-.047	.026	-.058
2.Up and down stairs	.046	.213*	-.104	-.007	.334**	.566**	.589**	.494**
3.Pick up slipper	.209	.281**	-.003	.078	.381**	.521**	.634**	.525**
4.Reach at eye level	-.003	.159	-.216*	-.083	.517**	.543**	.676**	.466**
5.Reach on tiptoes	.174	.135	-.253*	-.049	.374**	.611**	.654**	.557**
6.Stand on chair to reach	-.036	.095	-.163	-.010	.449**	.686**	.601**	.668**
7.Sweep the floor	.273*	.381**	.030	.030	.327**	.657**	.666**	.441**
8.Walk outside to nearby car	.046	-.071	.000	.132	.311**	.299**	.283**	.199
9.Get in/out of car	.036	.166	-.007	-.036	.334**	.458**	.461**	.376**
10.Walk across parking lot	.182	.116	-.023	.202	.348**	.489**	.540**	.322**
11.Up and down ramp	.052	.062	-.108	-.110	.114	.346**	.419**	.279**
12.Walk in crowded mall	.165	.015	.024	.184	.372**	.515**	.556**	.411**
13.Walk in crowd/bumped	.337**	.066	.103	.252*	.097	.250*	.294**	.169
14.Escalator holding rail	.224*	.013	-.233*	.050	.426**	.661**	.713**	.503**
15.Escalator not holding rail	.078	.124	-.057	-.116	.21	.433**	.435**	.467**
16.Walk on icy sidewalks	.259*	.214*	-.112	.140	.367**	.639**	.644**	.590**
Sitting time (1-4)	.186	-.010	-	.228*	-.194	-.115	-.187	-.220*

*p<0.05, **p<0.01

Further, overall activities-specific balance confidence significantly correlated with performance frequency in 13 activity items (*r*=0.279 to 0.668), 10 of which showed moderate-to-strong correlations. In these 13 activity items, there were also within-item significant correlations between frequency and confidence scores (*r*=0.246 to 0.724), seven of which showed moderate-to-strong correlations (Table 3).

**Table 3:** Correlations between activities-specific frequency and balance confidence

Correlations (Spearman’s rho)	ABC (0-100)																
	1	2	3	4	5	6	7	8	9	10	11	12	13	14	15	16	overall
Activities-specific frequency (0-4)																	
1. Walk around house	.190																-.058
2. Up and down stairs		.493**															.494**
3. Pick up slipper			.422**														.525**
4. Reach at eye level				.372**													.466**
5. Reach on tiptoes					.539**												.557**
6. Stand on chair to reach						.724**											.668**
7. Sweep the floor							.587**										.441**
8. Walk outside to nearby car								.246*									.199
9. Get in/out of car									.100								.376**
10. Walk across parking lot										.330**							.322**
11. Up and down ramp											.308**						.279**
12. Walk in crowded mall												.289**					.411**
13. Walk in crowd/bumped													.135				.169
14. Escalator holding rail														.386**			.503**
15. Escalator not holding rail															.521**		.467**
16. Walk on icy sidewalks																.467**	.590**

*p<0.05, **p<0.01

**Fig. 1:**
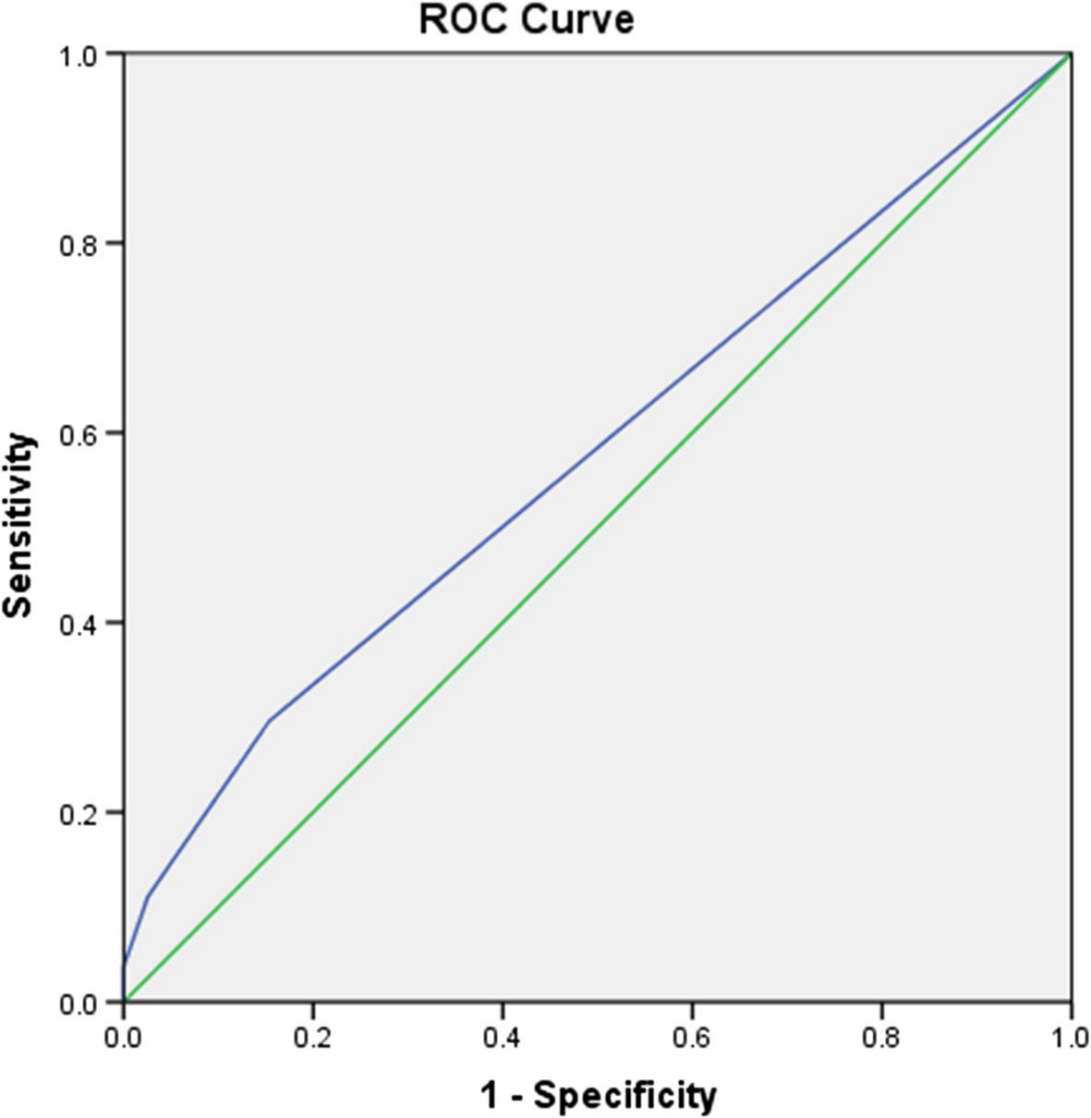
ROC curve for performance frequency in walking in crowds/bumped

Among all demographic and health-related characteristics, potential predictors of fall history included activities-specific performance frequency in two items: walking in crowded malls (item 12) and walking in crowds/bumped (item 13) (*p*<0.1). After entering both items into the multivariate regression model, only performance frequency of walking in crowds/bumped (item 13) entered in the final model via backward stepwise selection (odd ratio=3.310, 95%CI: 1.315-8.336, *p*<0.05). (Table 4). Then, using the ROC curve analysis, we observed that the cutoff point for this item was 0.50, showing a sensitivity of 34.8% and specificity of 86.2% for discriminating fall history; nonetheless, the prediction did not reach statistical significance (*p*=0.106) (Fig 1.).

**Table 4:** Backward Stepwise Multivariate Regression Model for fall history

Model	Variables	B	S.E.	Wald	df	Sig.	Exp(B)	95% C.I. for EXP(B)	
								Lower	Upper
Step 1a	ABCf12	.130	.309	.177	1	.674	1.139	.621	2.088
	ABCf13	1.084	.538	4.053	1	.044*	2.955	1.029	8.487
	Constant	-1.459	.351	17.328	1	.000	.232		
Step 2a	ABCf13	1.197	.471	6.455	1	.011*	3.310	1.315	8.336
	Constant	-1.38	.290	22.576	1	.000	.252		

a Variable(s) entered on step 1: ABCf12, ABCf13.

ABCf12: frequency of walking in crowded mall (item 12), ABCf13: frequency of walking in crowd/bumped (item 13)

*p<0.05

## Discussion

This study innovatively quantified activity-restrictions in elderly participants using the performance frequency of ABC activities. The main purpose of this study was to test the validity of using activities-specific performance frequency to assess fall risks among older adults. Results of this study suggested that activities-specific performance frequency can show difference among older adults, correlate a series of fall risk factors including functional balance/mobility, balance confidence, and sensitively detect fall history of older adults.

We found greater activity restrictions in participants with stroke when compared to normal and PD counterparts. Here, only the item of walking around the house showed comparable performance frequencies across all groups, meaning that 15 ABC activities showed lower frequencies in those with stroke. Further, only those with stroke showed total restrictions in four activities, including standing on a chair to reach, walking in crowds/bumped, taking escalator while not holding onto a railing, and walking on icy sidewalks, some of which were reported in previous studies [5, 26]. Meanwhile, the normal and PD groups showed comparable performance frequencies in all activity items except for taking the escalator while holding onto a railing, which was more frequent in the PD group. Upon observing the results for the items of taking the escalator while holding a rail and not holding, we observed no engagement in both activities for the stroke group, or occasional engagement in both activities for the normal group, and different engagement levels for the PD group (frequency median: 2 vs 0). Regarding the results for individuals with PD, we believe that they appeared because although these individuals generally keep on engaging in daily activities (which may often include taking an escalator) in a similar fashion as to how individuals with no neurological diseases do, they may have intentionally or subconsciously chosen to take the escalator in a safer manner (i.e., holding onto a railing) owing to their awareness of fall prevention.

Sitting behavior between groups differed from activity-restrictions. Here, the stroke group showed sitting time similar to those in the normal group and much higher than those in the PD group. In this regard, the stroke participants may have worked to avoid overly inactive lifestyles, but relatively poorer balance confidence and decreased functional balance/mobility likely impeded their ability to maintain normal activities-specific performance frequencies. In this study, PD participants with relatively good or near-to-normal mobility and balance confidence were able to maintain daily activities as normal. In this case, less sitting time may have indicated an adaptation strategy aimed at improving overall health.

Based on activities-specific performance frequency, sitting time, and other health-related characteristics among the participant groups, we propose that activity restrictions more directly rely on functional balance/mobility and balance confidence than sitting behavior. This is supported by previous studies showing that self-reported activity restrictions, but not sedentary behaviors, were correlated with both physical function and the fear of falling in older adults (of note, balance confidence is interchangeably used with fear of falling in this context [27–31]. Specifically, we found that performance frequency in most activities was moderate-to-strongly correlated with both functional balance/mobility and balance confidence, whereas sitting time was weakly associated only with balance confidence, which endorses the assumption as well.

Regarding performance frequency, most activities demonstrated moderate-to-strong correlations with TUG and FAM, but weak-to-moderate associations with FR. This could be because most ABC items are associated with dynamic balance and mobility tasks, as are TUG and FAM, versus more static balance related activities where base of support is more stable, as in FR. That is, it could be activity-specific in the correlations between physical function and activity restriction.

We also further explored whether it is activity-specific in the correlations between balance confidence and activity restrictions. However, we found that within-item correlations between activities-specific performance frequency and balance confidence were comparable or even slightly weaker than correlations between performance frequency and overall balance confidence. This indicates that it is more valuable to focus on overall balance confidence than on such confidence for individual activity items.

The sitting activity we utilized in the study differs from activities performed while standing in the ABC scale, and this can explain one of our results that sitting time showed non-significant correlation with performance frequency of most ABC activities. The weak correlation between sitting time and performance frequency of only three activities of the ABC scale might due to the linkage of balance confidence with them.

Regarding fall history, it showed weak correlations only with the performance frequency of walking in crowds/bumped and sitting time. Then, binary logistic regression identified the performance frequency of being bumped while walking in crowds as the only independent discriminator of fall history (odds ratio of 3.310). Based on ROC curve analysis, a cutoff point of 0.50 could be used to indicate occasionally or more often experiencing the action of being bumped when walking in crowds induces more than triples the risk of falling. However, the ROC model had inadequate diagnostic efficiency as it did not reach the significance level. Unlike other ABC activities, being bumped when walking in crowds tends to be an unexpected event. The insignificant correlation between its frequency and balance confidence found in this study may support the notion that the occurrence is not intentional. In spite of this, based on the finding, paying more attention to avoid being bumped in the crowd environment is suggested for fall prevention.

Meanwhile, neither age, sex, nor other health-related characteristics (e.g., balance confidence and physical functions) were identified as independent fall risk factors. This may be the result of convenience sampling and/or the relatively small sample size used in the study, which was also found to reduce representativeness of the demographic and health health-related characteristics among the normal elderly and those with disabilities in previous researches [32, 33]. This was the main limitation of this study.

The second limitation was the cross-sectional approach, which did not allow for the determination of causal relationships between activities-specific performance frequency and other health-related characteristics. Besides, activity restriction is thought to have dual effects on fall prevention, which cannot be identified via correlation or regression analyses. As the CARFS integrates the dual effects of activity restriction in calculating scores for the composite activities-specific risk of falling, it may be a better tool than the ABC-based activities-specific performance frequency alone when attempting to understand these dual effects. Moreover, sitting time was collected to learn the common sedentary behavior, but cannot reflect physical activity level such as sedentary level which is determined by the amount of physical activity [6]. Thus, results related to sitting time should be interpreted with caution.

## Conclusions

This study investigated activities-specific performance frequency in elderly participants, including those without neurological diseases, those with stroke, and those with PD. Those with stroke showed much lower activities-specific performance frequency in daily activities, thus extensive activity restrictions. We found that activities-specific performance frequency had stronger correlations with activities-relevant functional mobility than with balance confidence. Finally, the performance frequency of walking in crowds/bumped was the most sensitive predictor of falling risk among all that were tested.

## Supplementary Information

**Additional file 1.**

## Data Availability

The datasets used and/ or analysed during the current study are available from the corresponding author on reasonable request.

## List of Abbreviations

PD: Parkinson’s disease
ABC: Activities-Specific Balance Confidence
SAFE: Survey of Activities and Fear of Falling in the Elderly
TUG: timed up and go
ANOVA: one-way analysis of variance
CARFS: composite activities-specific risk of falls scale
FR: functional reach
FAM: Functional Assessment Measure
ROC: receiver operating characteristic
