# Supplementary material for: Activities-specific performance frequency can accurately detect fallers in elderly populations: an alternative method for quantifying activity restrictions
Source: BMC Geriatr. 2022 Mar 14;22:205. doi: 10.1186/s12877-022-02912-z (PMC8922773; doi:10.1186/s12877-022-02912-z)
Supplement: Supplementary file 1 — Additional file 1. [file 12877_2022_2912_MOESM1_ESM.docx]

**Appendix I.**

**Questionnaire on Activities-Specific performance frequency and balance confidence modified from the Activities-Specific Balance Confidence (ABC) scale**

| **0%** | **10** | **20** | **30** | **40** | **50** | **60** | **70** | **80** | **90** | **100%** |
| --- | --- | --- | --- | --- | --- | --- | --- | --- | --- | --- |
| No confidence Absolutely confident | | | | | | | | | | |
| Will definitely lose balance or instability Definitely not out of balance or unstable | | | | | | | | | | |

**How much confidence do you have when you finish the following activities?^**

**How often did you do the following activities in the past month?#**

| **Activity items** | **Balance confidence (0-100%)** | **Performance frequency (0-4)**  0：none (none in the past month)  1：occasionally(done in the past month)  2：sometimes (done weekly)  3：often (done daily)  4：very often  (done daily, with higher frequency than the normal) |
| --- | --- | --- |
| 1. Walk around house |  |  |
| 1. Up and down stairs |  |  |
| 1. Pick up slipper from floor |  |  |
| 1. Reach at eye level |  |  |
| 1. Reach on tiptoes |  |  |
| 1. Stand on chair to reach |  |  |
| 1. Sweep the floor |  |  |
| 1. Walk outside to nearby car |  |  |
| 1. Get in/out of car |  |  |
| 1. Walk across parking lot |  |  |
| 1. Up and down ramp |  |  |
| 1. Walk in crowded mall |  |  |
| 1. Walk in crowd/bumped |  |  |
| 1. Escalator holding rail |  |  |
| 1. Escalator not holding rail |  |  |
| 1. Walk on icy sidewalks |  |  |

^If you usually use walkers for activities or need help from others, please rate your confidence while still using these help; If you haven't done it, imagine how confident you can perform this activity without losing balance or maintaining stability under your existing conditions.

#：If the time after disease/ injury onset is less than one month. The time after onset is used to estimate the frequency.
